# Regional impact of updated guidelines on prevalence and distribution of blood pressure categories for hypertension in India: Results from the National Family Health Survey 4

**DOI:** 10.1016/j.ihj.2021.06.004

**Published:** 2021-06-12

**Authors:** Kartik Gupta, Vardhmaan Jain, Armaan Qamar, Aayush K. Singal, Sivasubramanian Ramakrishnan, Rajeev Gupta, Navkaranbir S. Bajaj

**Affiliations:** a Division of General Internal Medicine, Department of Medicine, Henry Ford Hospital, Detroit, MI, USA; b Department of Medicine, Cleveland Clinic, Cleveland, OH, USA; c Section of Interventional Cardiology, NorthShore Cardiovascular Institute, NorthShore University Health System, Evanston, IL, USA; d Department of Cardiology, All India Institute of Medical Sciences, New Delhi, India; e Eternal Heart Care Centre and Research Institute, Jaipur, India; f Division of Cardiovascular Disease and Comprehensive Cardiovascular Center, University of Alabama at Birmingham, AL, USA

**Keywords:** Hypertension, Prevalence, 2017 ACC/AHA, India, Epidemiology

## Abstract

**Introduction:**

In 2017, the American College of Cardiology/American Heart Association revised guidelines for diagnosis and management of hypertension in adults. The regional impact of the updated guidelines on the prevalence of hypertension in India is unknown.

**Methods:**

Data from nationally representative Indian households were analyzed to estimate the regional prevalence of hypertension according to the old and the new guidelines in men (age 18–54 years) and women (age 18–49 years). The old guidelines defined hypertension as a systolic blood pressure of ≥140 mmHg or diastolic blood pressure of ≥90 mmHg or treatment. The new guidelines define hypertension as a systolic blood pressure of ≥130 mmHg or diastolic blood pressure of ≥80 mmHg or treatment. We calculated the increase in the prevalence of hypertension among the states and union territories of India (hereafter “states”).

**Results:**

Among 679,712 participants (85.6% women), the median age was 31 years (interquartile range 24, 40) and was comparable among men and women (33 vs. 31 years, respectively). The overall weighted prevalence according to old and new guidelines was 18.5% (95% CI 18.2, 18.7) and 43.0% (95% CI 42.8, 43.3), respectively. There was a significant increase in hypertension prevalence, both among men and women, and across all regions. The northeast region of the country had the highest prevalence.

**Conclusion:**

The overall prevalence of hypertension significantly increases with the new compared to the old guidelines, however, the regional heterogeneity of prevalence of hypertension is maintained.

## 1 Introduction

India has a high prevalence of hypertension and cardiovascular mortality.^1^, ^2^, ^3^ High systolic blood pressure is the most important modifiable risk factor for cardiovascular diseases.^4^ Aggressive blood pressure reduction, beyond the previously recommended target, has incremental benefit in reducing cardiovascular risk.^5^ Subsequently, in 2017, the American College of Cardiology/American Heart Association lowered the cutoff to define hypertension and the goal of blood pressure treatment.^6^ This led to an increase in the prevalence of hypertension.^6^, ^7^, ^8^

The lower threshold to define and manage hypertension will help identify patients where early intervention can prevent cardiovascular events. Promotion of a healthy lifestyle and sustained blood pressure control from an early age can have huge cost-effect benefits, especially for a low- and middle-income country such as India.^9^ This requires updated knowledge of the regional prevalence of hypertension among young men and women.^10^ This study aimed to analyze the impact of new guidelines on the prevalence of hypertension among young across India.

## 2 Methods

The study was conducted following the Strengthening the Reporting of Observational Studies in Epidemiology (STROBE) guidelines (Supplement Table 1).

We analyzed data from the 2015–2016 National Family Health Survey-4 (NFHS-4).^11^ This survey was conducted in 29 states and 6 union territories of India (hereafter called “states”) by the Ministry of Health and Family Welfare, Government of India, and the data was managed by the International Institute for Population Sciences. This survey was done to evaluate mother and child-related health factors and oversampled women aged 15–49 years. The survey was funded by the United States Agency for International Development and the Ministry of Health and Family Welfare, Government of India. The data was collected using Computer Assisted Personal Interviewing on minicomputers from January 20, 2015, to December 4, 2016, and was available on request from the Demographic and Health Surveys website.

All participants gave written, informed consent before participation in the survey. We used publicly available de-identified data and approval from the Institutional Review Board was not required.

All fieldworkers collecting data were trained to accurately measure blood pressure. The field manual and data collection questionnaire is available online.^12^ The blood pressure was measured using a portable Omron blood pressure monitor, model HEM-8712. The participant was seated quietly for ≥5 min before measurement and was asked to avoid smoking, tea, or coffee during measurement. The participant was seated on a table in a relaxed position with feet flat on the ground. Three blood pressure readings were obtained at an interval of 5 min, preferably from the participant's left arm. To ensure the accuracy of data collection, field supervisors revisited a random subset of participants. The biomarker and other data were electronically sent to the Indian Institute of Population Sciences daily. Further details are given in the field manual available online.^12^ We used the mean of the second and the third in participants with three readings and the mean of the first and the second reading in participants with two readings. The final blood pressure was rounded off to the nearest whole number.

We excluded participants aged <18 years and those with pregnancy at the time of BP measurement. We excluded participants with systolic blood pressure less than or equal to diastolic blood pressure. We also excluded participants with systolic blood pressure <60 mmHg or >250 mmHg or diastolic blood pressure <30 mmHg or >250 mmHg due to biological implausibility. There were no exclusion criteria based on treatment for hypertension.

### 2.1 Guidelines

The Eighth Joint National Committee (JNC8) guidelines (hereafter “old guidelines”) define hypertension as either a systolic blood pressure ≥140 mmHg or diastolic blood pressure of ≥90 mmHg or treatment with antihypertensive medications.^13^ The 2017 ACC/AHA guidelines (hereafter “new guidelines”) define hypertension as either a systolic blood pressure ≥130 mmHg or diastolic blood pressure of ≥80 mmHg or treatment with antihypertensive medications. The new guidelines further define four categories for classification: normal (systolic blood pressure <120 and diastolic blood pressure <80 mmHg), Elevated (systolic blood pressure 120–129 and diastolic blood pressure <80 mmHg), Stage 1 hypertension (systolic blood pressure 130–139 or diastolic blood pressure 80–89 mmHg) and Stage 2 hypertension (systolic blood pressure ≥140 or diastolic blood pressure ≥90 mmHg).^6^ We excluded participants on anti-hypertensive medications while categorizing blood pressure.

### 2.2 Outcome

The main outcome of the study was to assess the national and regional impact of the 2017 ACC/AHA guidelines on the prevalence of hypertension in India.

### 2.3 Statistics

In this survey, 2-stage cluster sampling was used. The primary sampling unit was a village for a rural area or a census enumeration block in an urban area. This sampling was done using probability proportion to population, with the 2011 census population of India as the reference. Next, systematic random sampling was used to select households within each sampling unit. Data from men aged 15–54 years were collected from 15% of randomly selected households.

Systolic and diastolic blood pressure was defined as the average of the 2nd and the 3rd blood pressure reading. In patients with only 2 blood pressure readings, the average of the first 2 readings was taken. Household sampling weights were used for calculating the overall prevalence. Strata with singleton primary sampling units were treated as certainty units and contributed nothing to the standard error around the overall weighted prevalence.^14^ We grouped the states into regions to assess impact. Details are given in Supplement Methods.

Continuous variables were reported as medians with interquartile ranges (IQRs) and categorical variables were represented as counts with proportions. Chi-squared tests were used to identify the differences in hypertension prevalence with new guidelines and across different states. All statistical analyses were conducted in Stata version 14.2 (Stata Corp, College Station, TX, U.S.A.).

## 3 Results

Data from 679,712 participants were available for analysis (S1Supplement Figure 1). The median age of the study population was 31 years (IQR 24, 40); 85.5% were women. Three readings of systolic blood pressure and diastolic blood pressure were available in 97.7% of the study participants. Mean systolic blood pressure among men and women was 121 mmHg (IQR 113, 129) and 114 mmHg (106, 122), respectively. Mean diastolic blood pressure among men and women was 80 mmHg (IQR 74, 86) and 77 mmHg (IQR 71, 83), respectively.

The overall weighted prevalence according to the old guidelines was 18.5% (95% CI 18.2, 18.7). The overall weighted prevalence according to the new guidelines was 43.0% (95% CI 42.8, 43.3). There was significant heterogeneity in the overall prevalence across the region: the prevalence according to the new guideline ranged from 39.9% (95% CI 39.7, 40.1) in the central region to 51.8% (95% CI 51.5, 52.1) in the northeastern region (Supplement Table 2). The prevalence according to the old guidelines in the central and the northeastern region was 14.6% (95% CI 14.5, 14.8) and 23.1% (95% 22.8, 23.4), respectively.

**Fig. 1:**
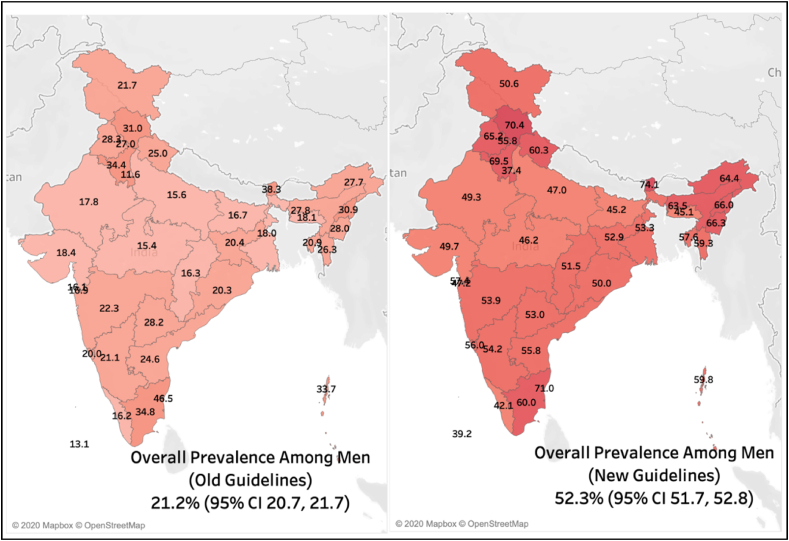
Heat map for the prevalence of hypertension among men according to old (Panel A) and new (Panel B) guidelines.

**Fig. 2:**
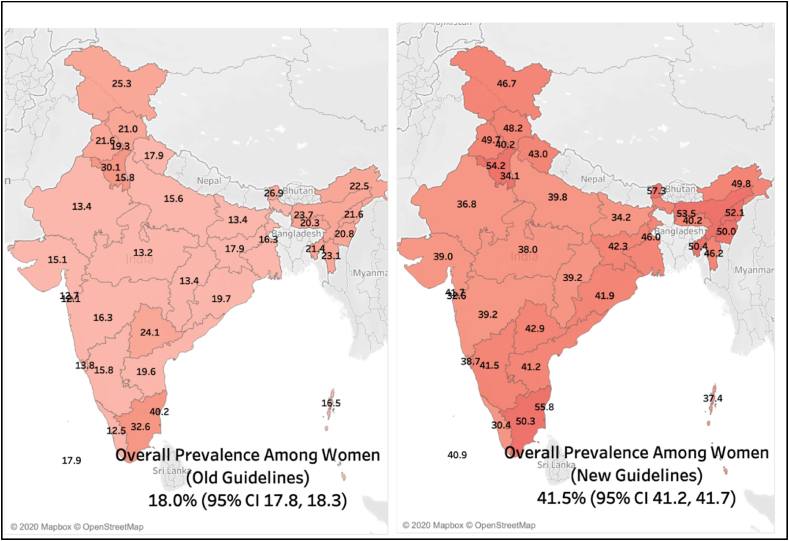
Heat map for the prevalence of hypertension among women according to old (Panel A) and new (Panel B) guidelines.

The overall weighted prevalence according to old and new guidelines among men was 21.2% (95% CI 20.7, 21.7) and 52.3% (95% CI 21.7, 52.8), respectively (Fig. 1A and B). The overall weighted prevalence according to old and new guidelines among women was 18.0% (95% CI 17.8, 18.3) and 41.5% (95% CI 41.2, 41.7), respectively (Fig. 2A and B).

**Fig. 3:**
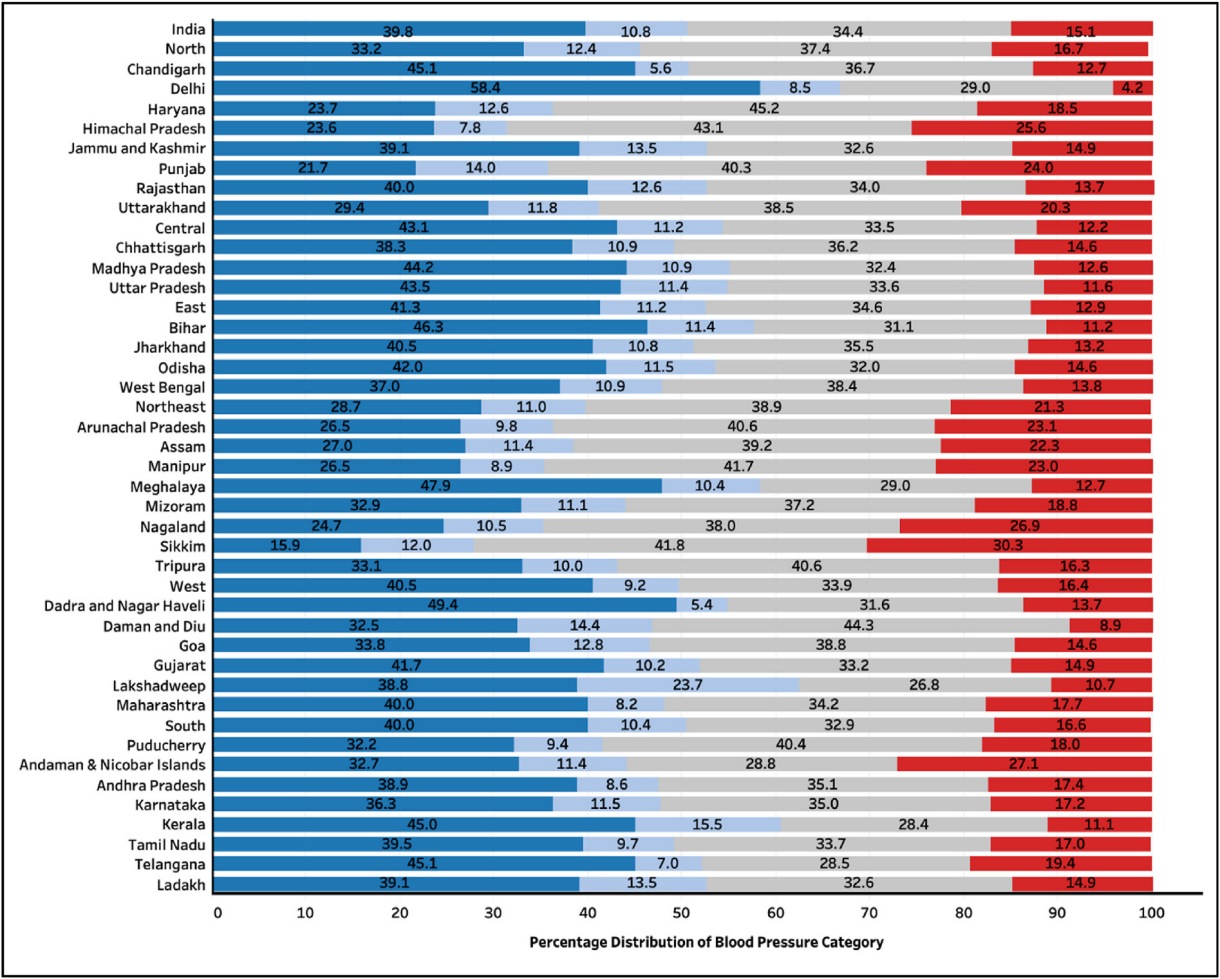
Distribution of blood pressure categories among men across states of India (n = 657,282). Dark Blue: Normal blood pressure, Light blue: elevated blood pressure, Grey: Stage 1 hypertension, Red: Stage 2 hypertension.

**Fig. 4:**
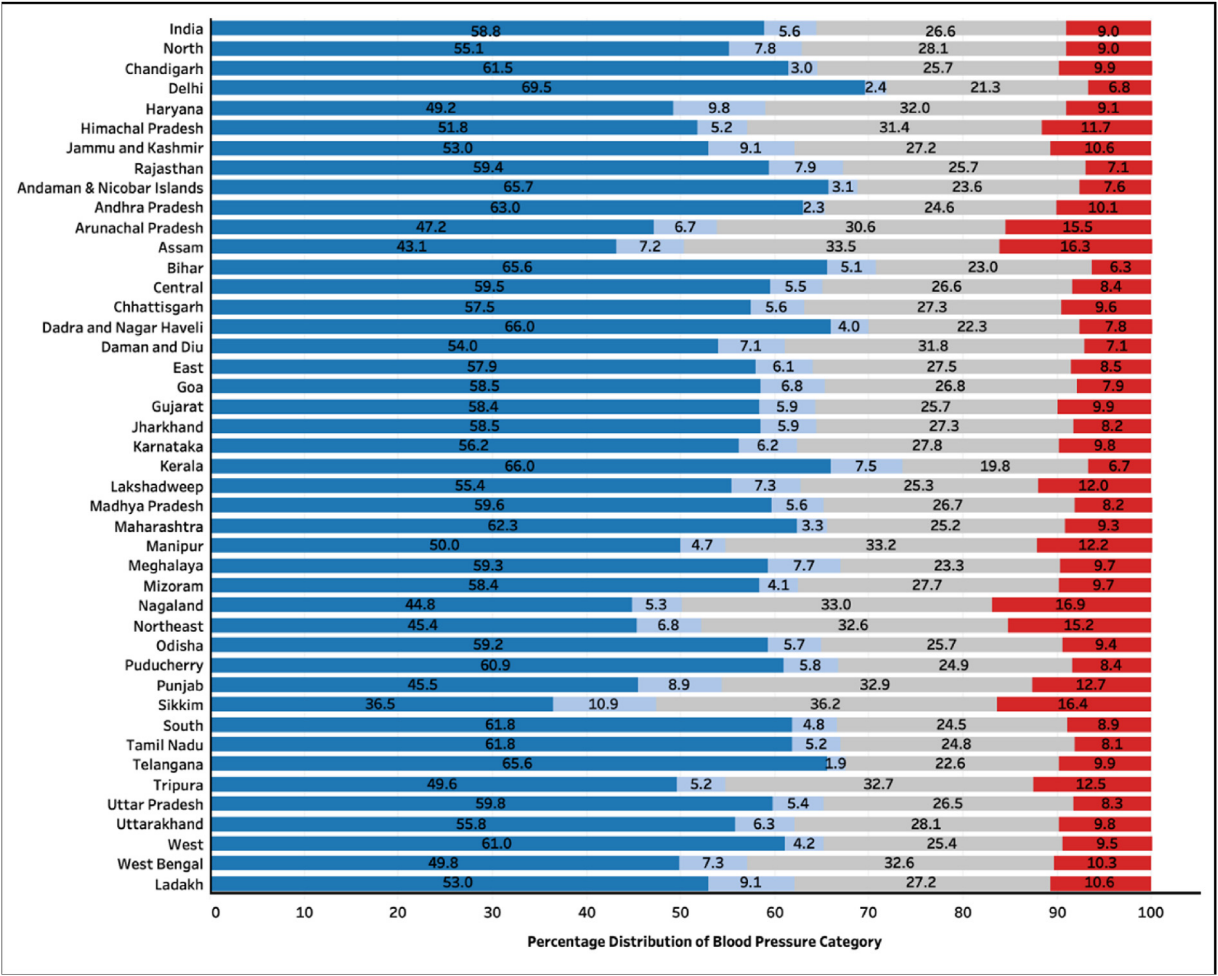
Distribution of blood pressure categories among women across states of India (n = 657,282). Dark Blue: Normal blood pressure, Light blue: elevated blood pressure, Grey: Stage 1 hypertension, Red: Stage 2 hypertension.

The distribution of BP categories across the four new stages of hypertension among participants not on any anti-hypertension medications (96.7% of the study population), is given in Fig. 3, Fig. 4. There were only 39.8% (95% CI 39.3, 40.3) men and 58.8% (95% CI 58.6, 59.1) women with normal blood pressure (Figs. 3 and 4).

## 4 Discussion

In this analysis of a survey population of Indian adults aged 18–54 years for men and 18–49 years for women, the overall prevalence of hypertension increased by 2.3 times from 18.5% to 43% with the new guidelines. Overall, less than 50% of men and less than 60% of women had normal blood pressure according to the new guidelines.

Large randomized control trials have suggested an incremental benefit of intensive blood pressure control to prevent adverse cardiovascular events.^5^ Consequently, in 2017, the new guidelines lowered the cut-off to define hypertension and set new goals for blood pressure management.^6^ There is an expected increase in the prevalence of hypertension, as seen in other countries such as the United States, China, and Bangladesh.^7^^,^^8^^,^^15^, ^16^, ^17^, ^18^ A study from a different large survey data from India reported an increase in prevalence from 21.8% to 52.3% with the new guidelines.^9^ Our analyses confirm these findings in another large survey dataset. This increase is because participants who previously had “prehypertension”, defined as a systolic blood pressure of 120–139 mmHg or diastolic blood pressure 80–89 mmHg according to the old guidelines, are now classified as either elevated blood pressure or stage 1 hypertension.^7^

An analysis from the Global Burden of Disease Study in 2016 showed a wide regional variation in the burden of cardiovascular diseases in India.^2^ To the best of our knowledge, this is the first time that the regional impact of new guidelines has been reported for India.NFHS-4collected data from participants aged 18–54 years and 85% were women. The lower prevalence according to the JNC8 guidelines in our current study as compared to other studies is likely due to enrollment of a younger population and oversampling of women.^1^^,^^19^^,^^20^ The overall prevalence in India, therefore, is expected to be higher.

National Health Policy of India in 2017 aims to reduce premature deaths due to cardiovascular causes by 25% and treating ≥80% of patients with hypertension by 2025 (NHP). Understanding regional heterogeneity in cardiovascular risk factors might help direct targeted and cost-effective interventions. Similar to previous studies, our study suggests a disproportionately high prevalence in the Northeast region.^19^^,^^21^ The exact reasons for this are unknown. Some of the plausible factors are a higher prevalence of tobacco smoking, a salt-rich diet, and the effects of higher altitude. Data for these risk factors were not available for analysis.

Besides conventional risk factors such as obesity, and smoking, high ambient air pollution, and dietary salt consumption are of special importance in India.^22^, ^23^, ^24^, ^25^ Currently, there is no data from India that suggests an independent association of air pollution with higher blood pressure in India.

At a national level, the government should invest in population screening and promotion of a healthy lifestyle, including but not limited to a low salt diet and regular physical exercise. At a local level, the primary health care centers and sub-centers can serve as valuable tools to deliver preventive health care and blood pressure management. Strategies for hypertension management can also help manage other cardiovascular risk factors such as dyslipidemia, diabetes mellitus, and obesity. It is important to note that the current Indian Guidelines for Hypertension-IV, published in 2019, do not endorse the lowered cut-off.^26^ We believe that a lower threshold to define hypertension can hasten discussion on cardiovascular risk reduction.

There are several limitations to our study. As discussed previously, there was oversampling of women and a relatively young age group. The results do not apply to the entire adult population. We aimed to assess the regional impact on the prevalence and did not analyze urban-rural differences. Current guidelines recommend taking 3 blood pressure readings on 2 different occasions.^6^ In the current survey, all readings were taking on a single occasion and not all participants had 3 readings. The guidelines also recommend that at the first visit, blood pressure be measured in both arms, and then the arm with the higher reading be used for subsequent readings.^6^ In this survey, the left arm was used by default unless the participant indicated otherwise. We did not have data to analyze other co-existing cardiovascular risk factors such as diabetes mellitus and dyslipidemia.

Currently, there is no longitudinal data from India to understand the benefit of more stringent blood pressure control. However, it is unlikely that the cardiovascular benefit would differ from the large, randomized control trials in other countries.^5^ Population-based intervention studies are required to identify the most cost-effective management strategies in a diverse country such as India.

## 5 Conclusion

In this analysis of a cross-sectional survey from India, we found a significant increase in the national and regional prevalence of hypertension according to the 2017 ACC/AHA guidelines. There is regional heterogeneity in the prevalence of hypertension.

## Funding

None.

## Declaration of competing interest

Dr. Qamar reports receiving institutional grant support from 10.13039/501100002973Daiichi-Sankyo, 10.13039/100000968American Heart Association, and fees for educational activities from the 10.13039/100005485American College of Cardiology, Society for Vascular Medicine, Society for Cardiovascular Angiography and Interventions, Janssen and Janssen, 10.13039/100004319Pfizer, Medscape, and Clinical Exercise Physiology Association. The other authors have no conflict of interest related to this work.
